# Long-term Evaluation of Bonding Performance of Universal Adhesives based on Different Dentinal Moisture Levels

**DOI:** 10.3290/j.jad.b3559027

**Published:** 2022-11-08

**Authors:** Rammon de Faria Nonato, Pedro Henrique de Aguiar Moreira, Daniella de Oliveira da Silva, Michel Wendlinger Cantanhe de Ferreira, Alessandra Reis, Andres Felipe Millan Cardenas, Alessandro D. Loguercio, Fabiana Suelen Figuerêdo de Siqueira

**Affiliations:** a MS Student, Department of Postgraduate Program in Dentistry, CEUMA University, São Luis, Maranhão, Brazil. Designed testing assembly, performed bond strength experiments, co-wrote paper.; b MS Student, Department of Postgraduate Program in Dentistry, CEUMA University, São Luis, Maranhão, Brazil. Designed testing assembly, performed bond strength experiments.; c PhD Student, Department of Postgraduate Program in Dentistry, CEUMA University, São Luis, Maranhão, Brazil. Designed testing assembly, silver nitrate uptake test, proofread the manuscript.; d MS Student, Department of Restorative Dentistry, State University of Ponta Grossa, Ponta Grossa, Paraná, Brazil. Designed testing assembly, performed scanning electron microscopy, co-wrote paper, proofread the manuscript.; e Professor, Department of Restorative Dentistry, State University of Ponta Grossa, Ponta Grossa, Paraná, Brazil. Co-wrote paper, provided consulting for statistical analysis, contributed substantially to the discussion.; f Professor, Department of Postgraduate Program in Dentistry, CEUMA University, São Luis, Maranhão, Brazil. Research idea, designed testing assembly, contributed substantially to the discussion, co-wrote paper, proofread the manuscript; g Professor, Department of Restorative Dentistry, State University of Ponta Grossa, Ponta Grossa, Paraná, Brazil. Provided consulting for statistical analysis, contributed substantially to the discussion, proofread the manuscript.; h Professor, Department of Postgraduate Program in Dentistry, CEUMA University, São Luis, Maranhão, Brazil. Research idea, designed testing assembly, contributed substantially to the discussion, co-wrote paper, proofread the manuscript.

**Keywords:** universal adhesive, dentin, microtensile bond strength, nanoleakage, water storage, wet-bonding.

## Abstract

**Purpose:**

To evaluate the microtensile bond strength (μTBS) and silver nitrate uptake (SNU) of three universal adhesives used in etch-and-rinse (ER) and self-etch (SE) modes on dry, wet, and oversaturated dentin surfaces after 24 h and 1 year of water storage. The morphology of the hybrid layer (MHL) and the degree of conversion (DC) were also evaluated.

**Materials and Methods:**

Human molars were divided into 36 groups according to combinations of the following variables: (i) universal adhesives (Ambar Universal APS [AMB], Prime&Bond Active [PBA], Scotchbond Universal Adhesive [SBU]), (ii) adhesive strategies (ER or SE), (iii) moisture level (dry, wet, or oversaturated dentin surface), and (iv) storage time (24 h or 1 year). After restoration, the specimens were sectioned into resin-dentin sticks and tested for μTBS and SNU according to storage time. For MHL, the specimens were sectioned and evaluated after 24 h using SEM. DC was evaluated using FTIR. ANOVA and Tukey’s test were used for statistical analyses (5%).

**Results:**

When 24-h vs 1-year data were compared, there was a significant decrease in μTBS and an increase in SNU values for the majority of experimental groups (p < 0.0001). On dry (ER) and oversaturated (ER and SE) dentin, AMB showed higher μTBS than did PBA (p < 0.00001). No significant decrease in μTBS was observed when universal adhesives were applied in the SE mode to dry dentin (p > 0.05). Regarding SNU, at all moisture levels, AMB showed lower SNU values than SBU (p < 0.001). Regarding MHL, SBU showed several imperfections when applied to oversaturated dentin in comparison with AMB and PBA. Regarding DC, when dentin was kept dry or was oversaturated, AMB showed a higher DC than PBA (p < 0.0001).

**Conclusion:**

The behavior of the different universal adhesives evaluated did not vary when applied to wet or dry dentin. However, the results with oversaturated dentin were dependent on the universal adhesive. Independent of the moisture level and the universal adhesive evaluated, significant degradation of the bonding properties occurred after 1 year of water storage, with the exception of universal adhesives applied to dry dentin in the SE strategy.

The evolution of adhesives has resulted in more simplified and less technique-sensitive materials, and has consequently improved application techniques.^21,46^ Contemporary adhesives are classified into two groups based on the mechanism of bonding to the tooth substrate: etch-and-rinse and self-etch.^46^

It is widely known that the permeability of demineralized dentin achieved with the etch-and-rinse technique is crucial for ensuring an adequate hybrid layer.^22^ After phosphoric acid etching, if the dentin is over-dried, the collagen fibrils might collapse, impairing the diffusion of monomers and consequently decreasing the bond strength.^45^ Thus, maintaining a moist substrate is recommended, because the remaining water keeps the collagen fibrils upright, thereby improving adhesive infiltration.^27^ On the other hand, the presence of water in self-etch adhesives is indispensable to ensure ionization of the functional monomers and hence make the adhesive acidic.^43^ Therefore, several studies have indicated that self-etch adhesives need to be applied to dry instead of moist dentin.^1,5,40,42^

However, obtaining standardized surface wetness is difficult because overwet and dry regions may exist on the same tooth surface and affect the bond strength of the etch-and-rinse and self-etch adhesives.^5,22,27,40,42,45^ It is even more complicated in the case of different walls in complex cavities (eg, MOD).^36^ Excess water on the dentin surface can interfere with the degree of polymerization of the adhesive.^39^ Furthermore, the collagen network swells and the interfibrillar space decreases, affecting adhesive infiltration into the demineralized dentin.^18^

Recently, a new generation of adhesives, known as universal adhesives, has been introduced on the market.^25^ They can be employed in etch-and-rinse (ER) or self-etch (SE) mode associated with phosphoric acid, making the procedure more versatile.^19,48^ The majority of universal adhesives available on the market contain 10-methacryloyloxydecyl dihydrogen phosphate (10-MDP), which promotes chemical adhesion to hydroxyapatite, creating a stable hybrid layer.^20,47^ However, to keep 10-MDP ionized, water is a necessary factor in the adhesive composition.^43^ Thus, several manufacturers claim that the adhesives can be applied at varying moisture levels.

Nevertheless, conflicting results have been obtained when different universal adhesives were applied to dry, wet, or overwet dentin.^13,14,28,41,43^ In addition, since excess water has been considered a potential cause of debonding in dentin,^3^ it is important to evaluate the long-term bonding properties of these adhesives to dentin. To the best of our knowledge, this is because the different dentin moisture conditions for universal adhesives were evaluated only immediately or in the short term.^13,14,28,36^

Therefore, this study aimed to evaluate the microtensile bond strength (μTBS) and silver nitrate uptake (SNU) of universal adhesives on dry, wet, and oversaturated dentin surfaces tested after 24 h and 1 year of water storage. In addition, the degree of conversion and morphology of the hybrid layer of universal adhesives on dry, wet, and oversaturated dentin surfaces were evaluated after 24 h. The null hypotheses tested were: (1) different moisture levels (dry, wet, and oversaturated) of the dentin surface do not affect the μTBS or SNU of the universal adhesives when applied using ER and SE strategies and evaluated after (2) 24 h or 1 year of water storage. (3) The different moisture levels (dry, wet, and oversaturated) of the dentin surface did not affect the degree of conversion.

## MATERIALS AND METHODS

### Tooth Selection and Preparation

**Fig 1:**
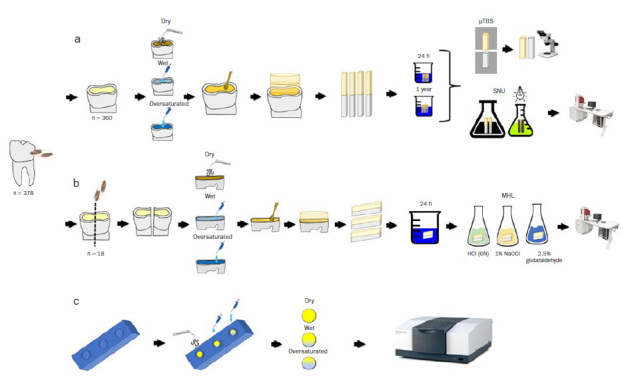
Schematic illustration of the experimental design. a. Microtensile bond strength and silver nitrate uptake. The occlusal third of the crown was removed from all the teeth using a diamond saw. After exposing the dentin surface, polishing, and smear layer standardization, all teeth were randomized according to moisture-level groups (dry, wet, and oversaturated). Distilled water was applied using a micropipette to simulate moisture levels. Subsequently, universal adhesives were applied, and the teeth were restored with composite resin. Bonded sticks were obtained and tested after 24 h or 1 year of water storage. After storage, the bonded sticks were tested for microtensile bond strength, failure mode, and silver nitrate uptake. b. Hybrid-layer morphology observation. After standardizing the dentin surface, two specimens were obtained from the each tooth and allocated to different surface moistures. The adhesives were then applied and the teeth restored. Bonded slices were obtained and examined using SEM. c. Degree of conversion measurements carried out using real-time Fourier transformed infrared spectroscopy. The universal adhesives were transferred to a plastic receptacle and evaluated before and after polymerization. Distilled water was added to the adhesive solution to simulate different moisture levels.

Three hundred seventy-eight (378) human molars were used in this study. The teeth were collected after approval by the ethics committee of the State University of Ponta Grossa, PR, Brazil (#3.115.355). They were disinfected with 0.5% chloramine and stored in distilled water until use. The occlusal third of the crown was removed from all teeth using a diamond saw under water-cooling in a cutting machine (Isomet, Buehler; Lake Bluff, IL, USA) to obtain a flat dentin surface (Fig 1). The enamel present along the margins was removed using a diamond bur (# 3195, KG Sorensen, Barueri, Brazil) in a high-speed handpiece under cooling. To confirm the absence of enamel on the dentin surface, careful examination was performed under a stereomicroscope (Olympus SZ40, Tokyo, Japan) at 30X magnification. The exposed dentin surfaces were further polished on wet, #600-grit silicon-carbide abrasive paper (SiC) for 30 s to standardize the smear layer.

### Experimental Design

Three hundred sixty (360) teeth were randomly divided into 36 groups (n = 10 dentin specimens) based on combinations of the main variables: 1. universal adhesives Ambar Universal APS (AMB, FGM; Joinville, SC, Brazil), Prime & Bond Active (PBA, Dentsply Sirona; Konstanz, Germany, also known as Prime & Bond Universal in some countries), or Scotchbond Universal (SBU, 3M Oral Care; St Paul, MN, USA, also known as Single Bond Universal in some countries); 2. adhesive strategies (etch-and-rinse [ER] or self-etch [SE] mode); and 3. surface moisture levels (dry, wet, or oversaturated) (Fig 1). In addition, as half of the specimens obtained for each tooth were randomly divided and tested after 24 h or 1 year of storage in water at 37°C, time was considered the fourth variable. More details regarding product information and application modes are provided in Table 1. Additionally, the other 18 teeth were used to evaluate the morphology of the hybrid layer using scanning electron microscopy (SEM).

**Table 1:** Adhesives, composition, groups, and application mode

Adhesives/batch number	Manufacturer	Composition	Dentin surface	Application mode*	
				Etch-and-rinse	Self-etch
Ambar Universal APS (AMB) 121217	FGM; Joinville, SC, Brazil	10-MDP, methacrylic monomers, photo-initiator, silica nanoparticles, ethanol, co-initiators, and stabilizers	Dry	Apply 37% phosphoric acid for 15 s. Rinse for 30 s. Air dry for 30 s. Apply adhesive as for the self-etch mode.	The dentin was kept dry for 30 s before adhesive application. Apply two layers with a microbrush for 20 s (10 s for each layer). Evaporate the adhesive solvent by using gentle air for 10 s. Light cure for 10 s
			Wet	Apply 37% phosphoric acid for 15 s. Rinse for 30 s. Air dry for 5 s. Apply 2.5 µl of distilled water with a micropipette. Apply adhesive as for the self-etch mode.	On a dry dentin surface, apply 2.5 µl of distilled water with a micropipette. Apply two layers of adhesive with a microbrush for 20 s (10 s for each layer). Evaporate the adhesive solvent with a gentle air stream for 10 s. Light cure for 10 s.
			Oversaturated	37% phosphoric acid for 15 s. Rinse for 30 s. Air dry for 5 s. Apply 4.5 µl of distilled water with a micropipette. Do not dry. Apply adhesive as for the self-etch mode.	On a dry dentin surface, apply 4.5 µl of distilled water with a micropipette. Do not dry. Apply two layers of adhesive with a microbrush for 20 s (10 s each layer). Evaporate the adhesive solvent with a gentle air stream for 10 s. Light cure for 10 s at 1400 mW/cm^2^.
Prime & Bond Active (PBA) 1706000711	Dentsply Sirona; Konstanz, Germany	Bisacrylamide 1, 10-MDP, bisacrylamide 2, 4-(dimethylamino) benzonitrile, dipentaerythritol pentacrylate phosphate (PENTA), 2-propanol, water	Dry	Apply 37% phosphoric acid for 15 s. Rinse for 30 s. Air dry for 30 s. Apply adhesive as for the self-etch mode.	The dentin was kept dry for 30 s before adhesive application. Apply the adhesive to the entire preparation with a microbrush and rub it in for 20 s. Blow a gentle air stream over the liquid for at least 5 s. Light cure for 10 s at 1400 mW/cm^2^.
			Wet	37% phosphoric acid for 15 s. Rinse for 30 s. Air dry for 5 s. Apply 2.5 µl of distilled water with a micropipette. Apply adhesive as for the self-etch mode.	On a dry dentin surface, apply 2.5 µl of distilled water with a micropipette. Apply the adhesive to the entire preparation with a microbrush and rub it in for 20 s. Blow a gentle air stream over the liquid for at least 5 s. Light cure for 10 s at 1400 mW/cm^2^.
			Oversaturated	37% phosphoric acid for 15 s. Rinse for 30 s. Air dry for 5 s. Apply 4.5 µl of distilled water with a micropipette. Do not dry. Apply adhesive as for the self-etch mode.	On a dry dentin surface, apply 4.5 µl of distilled water with a micropipette. Do not dry. Apply two layers of adhesive with a microbrush for 20 s (10 s for each layer). Let the adhesive solvent evaporate by using a gentle air stream for 10 s. Light cure for 10 s at 1400 mW/cm^2^.
Scotchbond Universal Adhesive (SBU) 1926600462	3M Oral Care; St Paul, MN, USA	10-MDP, dimethacrylate resins, HEMA, methacrylate-modified polyalkenoic acid copolymer, nanofiller, ethanol, water, initiators, silane	Dry	Apply 37% phosphoric acid for 15 s. Rinse for 30 s. Air dry for 30 s. Apply adhesive as for the self-etch mode.	The dentin was kept dry for 30 s before adhesive application. Apply the adhesive to the entire preparation and leave undisturbed for 20 s. Direct a gentle air stream over the liquid for about 5 s until it no longer moves and the solvent evaporates completely. Light cure for 10 s at 1400 mW/cm^2^.
			Wet	37% phosphoric acid for 15 s. Rinse for 30 s. Air dry for 5 s. Apply 2.5 µl of distilled water with a micropipette. Blot-dry with absorbent paper. Apply adhesive as for the self-etch mode.	On a dry dentin surface, apply 2.5 µl of distilled water with a micropipette. Apply the adhesive to the entire preparation and leave undisturbed for 20 s. Direct a gentle air stream over the liquid for about 5 s until it no longer moves and the solvent evaporates completely. Light cure for 10 s at 1400 mW/cm^2^.
			Oversaturated	37% phosphoric acid for 15 s. Rinse for 30 s. Air dry for 5 s. Apply 4.5 µl of distilled water with a micropipette. Do not dry. Apply adhesive as for the self-etch mode.	On a dry dentin surface, apply 4.5 µl of distilled water with a micropipette. Do not dry. Apply the adhesive to the entire preparation and leave undisturbed for 20 s. Direct a gentle air stream over the liquid for about 5 s until it no longer moves and the solvent evaporates completely. Light cure for 10 s at 1400 mW/cm^2^.

*The materials were applied according to the respective manufacturer’s recommendations. MDP: methacryloyloxydecyldihydrogen phosphate; HEMA: 2-hydroxyethyl methacrylate.

### Sample Size Calculation

For μTBS, the sample size was determined by considering the μTBS of SBU to dentin. The mean and standard deviation of SBU reported in the literature was 53.6 ± 5.5 MPa.^4,19,31^ To detect a difference of 8 MPa between the tested groups using a two-sided test and a significance level and power of 5% and 80%, respectively, the minimum sample size was n=8 teeth per group. For SNU, the sample size was determined by considering the SNU values of SBU on wet dentin. The mean and standard deviation of SBU reported in the literature were 6.6 ± 2.0 (%).^20,31,32^ To detect a difference of 3.3 among the tested groups using a two-sided test and a significance level and power of 5 and 80%, respectively, the minimum sample size was n=8 teeth per group. Both the sample sizes were calculated using a website (www.sealedenvelope.com).

### Restorative Procedures

In ER mode, after acid etching with 37% phosphoric acid gel (Condac, FGM; Joinville, SC, Brazil), the dentin surfaces were rinsed with distilled water for 20 s and air dried for 5 s with oil-free compressed air. In the dry-surface group, the dentin surface was air dried for 30 s.^26,32^ In the wet-surface group, 2.5 μl of distilled water were applied onto each dentin surface with a micropipette (Pipetman; Gilson, NY, USA) (Fig 1).^26,32^ Each dentin surface was wetted with an additional 4.5 μl of distilled water to simulate oversaturation (Fig 1).^26,32^

In SE mode, after polishing, the dentin surfaces were rinsed with distilled water for 20 s and air dried for 5 s with oil-free compressed air. In the dry-surface group, the dentin surface was air dried for 30 s. ^26,32^ For the wet and oversaturated groups, the dentin surfaces were treated like that of the ER group, as described above. The adhesives were applied according to the respective manufacturer’s instructions (Table 1). Furthermore, composite resin buildups (Opallis, FGM; Joinville, SC, Brazil) were placed in layers of 2 mm each, and each increment was light cured for 40 s (1400 mW/cm^2^, Valo, Ultradent; South Jordan, UT, USA). A single trained operator performed all the procedures (Fig 1).

After the restored teeth were stored in distilled water at 37°C for 24 h, the specimens were placed in a cutting machine (Isomet, Buehler; Lake Bluff, IL, USA), cut first longitudinally, then a second cut was made perpendicular to the first to obtain bonded sticks with a cross-sectional area of approximately 0.8 mm^2^. These were measured using digital calipers (Digimatic Caliper, Mitutoyo; Tokyo, Japan) to calculate the bond strength in MPa. The number of bonded sticks that debonded prematurely during specimen preparation were recorded for each tooth (Table 2).

**Table 2:** Number of specimens (%) according to fracture mode

Moisture level	Time	AMB						PBA						SBU					
		ER			SE			ER			SE			ER			SE		
		A/M	C	PF	A/M	C	PF	A/M	C	PF	A/M	C	PF	A/M	C	PF	A/M	C	PF
Dry	24 h	102 (100)	0 (0)	0 (0)	110 (96)	0 (0)	4 (4)	122 (98)	0 (0)	2 (2)	103 (97)	0 (0)	3 (3)	120 (97)	0 (0)	4 (3)	115 (97)	0 (0)	4 (3)
	1 year	107 (98)	0 (0)	2 (2)	112 (100)	0 (0)	0 (0)	118 (94)	2 (2)	6 (4)	106 (95)	0 (0)	6 (5)	120 (96)	0 (0)	5 (4)	114 (95)	0 (0)	6 (5)
Wet	24 h	115 (99)	0 (0)	1 (1)	124 (99)	0 (0)	1 (1)	108 (100)	0 (0)	0 (0)	114 (97)	0 (0)	4 (3)	115 (97)	0 (0)	3 (3)	110 (99)	1 (1)	0 (0)
	1 year	104 (96)	0 (0)	4 (4)	110 (96)	0 (0)	4 (4)	104 (98)	0 (0)	2 (2)	116 (100)	0 (0)	0 (0)	120 (100)	0 (0)	0 (0)	113 (97)	0 (0)	4 (3)
Over-saturated	24 h	120 (100)	0 (0)	0 (0)	106 (96)	0 (0)	4 (4)	120 (100)	0 (0)	0 (0)	120 (100)	0 (0)	0 (0)	107 (96)	2 (2)	2 (2)	105 (96)	1 (1)	3 (3)
	1 year	108 (98)	0 (0)	2 (2)	120 (98)	0 (0)	2 (2)	122 (100)	0 (0)	0 (0)	100 (96)	0 (0)	4 (4)	117 (97)	0 (0)	4 (3)	102 (97)	0 (0)	3 (3)

Abbreviations: A/M: adhesive/mixed fracture mode; C: cohesive fracture mode; PF: premature failures.

The bonded sticks were assigned to different tests: silver nitrate uptake, 3 bonded sticks per tooth and from each experimental condition were tested after 24 h or 1 year of water storage; the remaining bonded sticks underwent µTBS testing after 24 h or 1 year of water storage. Water was changed monthly (Fig 1).

### Microtensile Bond Strength Test (μTBS)

After 24 h or 1 year of water storage, the bonded sticks were attached to a modified Geraldeli device^24^ using cyanoacrylate resin. They were then subjected to a tensile force in a universal testing machine (Katros Dinamometros; Cotia, SP, Brazil) at a crosshead speed of 0.5 mm/min until bond failure occurred. μTBSs were calculated by dividing the load at failure by the cross-sectional bonding area.

The failure mode of each bonded stick was observed using a digital microscope (Olympus SZ40; Tokyo, Japan) and classified as cohesive (C; failure exclusively within dentin or resin), adhesive/mixed (A/M; adhesive or mixed failure within any of the bonded substrates, Table 2). Pre-test failures (PF) were included in the tooth mean with a value of 4.0 MPa for statistical anaylsis.^26^

### Silver Nitrate Uptake (SNU)

**Fig 2:**
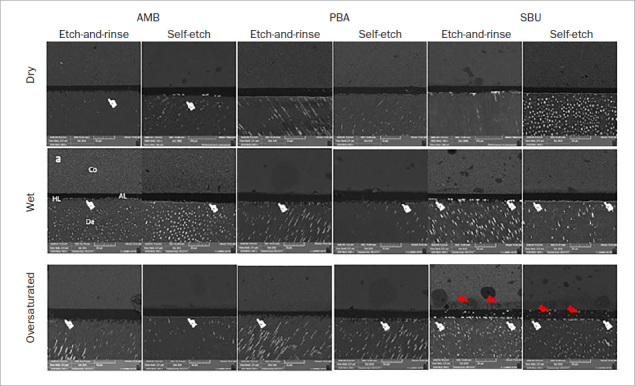
Representative backscattered SEM images (600X magnification) of silver nitrate uptake of the experimental group after 1 year of water storage. Independent of the dentin moisture level, AMB and PBA showed reduced SNU at the resin-dentin interface (white pointer). After 1 year of storage, SBU showed an increase of SNU and the presence of water bubbles (red pointer) when dentin was kept oversaturated. Co: resin composite; AL: adhesive layer; HL: hybrid layer; De: dentin.

Three bonded sticks per tooth from each storage period were not used in the µTBS test. First, all resin-dentin bonded sticks selected for this test were coated with two layers of nail varnish applied to within 1 mm of the bonded interfaces. All resin-dentin bonded sticks were placed in an ammoniacal silver nitrate solution in darkness for 24 h, rinsed in distilled water, and immersed in a photo-developing solution for 8 h under fluorescent light.^37^ The specimens were polished with 2500-grit SiC paper and 1- and 0.25-mm diamond paste (Buehler; Lake Bluff, IL, USA). After ultrasonic cleaning and air drying, the specimens were mounted on stubs, dried overnight in a desiccator, and coated with carbon gold. The silver penetration levels at the resin-dentin interface of each specimen were analyzed using a field-emission scanning electron microscope in backscatter mode (VEGA 3 TESCAN, Shimadzu; Tokyo, Japan) (Fig 2).

Three images of each bonded stick were captured: the first at 0.3 mm to the right of the center, the second at 0.3 mm to the left of the center, and the third image at the center. ImageJ software was used to determine the relative percentage of silver nitrate uptake along the adhesive and hybrid layers in each specimen.^10^

### Morphology of the Hybrid Layer (MHL)

Eighteen teeth were used in this part of the study. The dentin surface was prepared as previously described, and the crown was longitudinally sectioned in a buccal-to-lingual direction using a water-cooled low-speed diamond saw (Isomet) to obtain two specimens from each tooth. Specimens from the same teeth were used to evaluate the adhesives applied using different adhesive strategies. After allocation, each dentinal specimen was assigned one of 3 surface-moisture groups (dry, wet, or oversaturated dentin surfaces) and restored as previously described (Fig 1).

Restored teeth were cut longitudinally to obtain bonded slices with an area of approximately 1.0 mm^2^. Subsequently, each sample was polished using wet SiC paper (grit #1500, 2000, and 2500). After ultrasonic cleaning, the specimens were demineralized in HCl (6N) for 30 s and deproteinized in 1% NaOCl for 10 min to remove the hybrid layer. Then, the specimens were fixed in 2.5% glutaraldehyde in 0.1 M sodium cacodylate buffer at pH 7.4 for 12 h at 4ºC, rinsed with 20 ml of 0.2 M sodium cacodylate buffer at pH 7.4 for 1 h, and dehydrated in ascending grades of ethanol: 25% (20 min), 50% (20 min), 75% (20 min), 95% (30 min), 100% (60 min) (Fig 1).^12,30^

Following preparation, the specimens were mounted on stubs and sputter-coated with gold/palladium in a vacuum evaporator (SCD 050; Balzers; Schaan, Liechtenstein). The entire surface was examined using a scanning electron microscope (MIRA3 LM; Tescan Orsay; Warrendale, PA, USA). Three photomicrographs of representative surface areas were obtained at a magnification of 1800X.

### Degree of Conversion

The degree of conversion (DC) was evaluated using a real-time Fourier transform infrared spectroscope (IRPrestige21; Shimadzu; Tokyo, Japan) equipped with an attenuated total-reflectance device. Briefly, 10 μl of each adhesive was transferred to a small plastic receptacle and air dried for 30 s to remove the solvent. To simulate the dry, wet, or oversaturated dentin, 0.0 μl (dry), 2.5 μl (wet) or 4.5 μl (oversaturated) of distilled water were added to the adhesive solution (Table 1) (Fig 1). The material was then placed on a diamond crystal. Spectra were captured before and after the polymerization process. The degree of double bond conversion was obtained by considering the height of the absorption band (% of absorbance) corresponding to the C=C aliphatic bond at 1638 cm^-1^, and as an internal standard, the height of the absorption band (% of absorbance) corresponding to the C=C aromatic bond at 1609 cm^-1^. Each test was performed in triplicate.

### Statistical Analysis

The μTBS and SNU data of all the bonded sticks from the same hemitooth were averaged for statistical purposes. Therefore, the experimental unit in this study was the “hemi-tooth”. After evaluating the normality (Kolmogorov-Smirnov test) and equality of variances (Bartlett test), the μTBS (MPa) and SNU (%) data were subjected to four-way repeated measures ANOVA (adhesive vs adhesive strategies vs moisture level vs storage time) and Tukey’s test. For the DC test, after evaluating the normality (Kolmogorov-Smirnov test) and equality of variances (Bartlett test), a two-way ANOVA (adhesive vs moisture level) was applied, followed by Tukey’s test. The level of significance was set at 5%. All analyses were performed using SPSS (version 17.0; IBM; Armonk, NY, USA).

## RESULTS

### Microtensile Bond Strength (μTBS)

The mean cross-sectional area of the tested resin-dentin sticks was 0.80 ± 0.05 mm^2^. Approximately 10–14 resin-dentin sticks per hemi-tooth were obtained, including premature failures. The most common failure pattern was the adhesive/mixed in all experimental groups (Table 2). Only the triple cross-product interaction was significant (adhesive vs moisture level vs storage time, Table 3; p < 0.00001). In addition, the main factors adhesive (p = 0.001), moisture level (p = 0.0001), and storage time (p = 0.0001) were significant. The main factor adhesive strategy was not significant (p = 0.12).

**Table 3:** Means and standard deviations of microtensile bond strength (MPa) for all experimental groups

Moisture level	Time	AMB		PBA		SBU	
		ER	SE	ER	SE	ER	SE
Dry	24 h	45.2 ± 3.9 a,b	44.3 ± 3.2 b	44.1 ± 4.7 b	45.4 ± 3.2 a,b	43.2 ± 3.7 b	49.2 ± 3.6 a
	1 year	40.2 ± 4.1 c	40.1 ± 3.1 b,c	33.8 ± 4.2 d	39.2 ± 3.2 b,c	35.2 ± 3.8 c	44.1 ± 3.2 a,b
Wet	24 h	45.1 ± 4.6 a,b	46.2 ± 4.5 a,b	45.9 ± 4.6 a,b	43.9 ± 5.1 b	49.7 ± 4.4 a	48.4 ± 4.7 a
	1 year	38.0 ± 4.5 c	38.4 ± 3.3 c	35.1 ± 4.9 c,d	35.3 ± 5.4 c,d	39.8 ± 4.6 b,c	40.3 ± 4.4 b,c
Oversaturated	24 h	49.1 ± 5.1 a	47.4 ± 5.4 a	37.1 ± 4.2 c	39.8 ± 4.3 b,c	31.1 ± 3.8 d	33.3 ± 2.6 d
	1 year	39.0 ± 4.7 b,c	39.5 ± 4.1 b,c	33.3 ± 5.4 d	33.5 ± 4.6 d	24.1 ± 4.1 e	25.1 ± 5.4 e

Different letters indicate statistically significantly different means (four-way ANOVA and Tukey’s test; p < 0.05).

After 24 h, no significant difference in μTBS was observed for any of the adhesives evaluated when the dentin was dry or wet (p > 0.05). The exception was SBU used in ER mode. However, when dentin was oversaturated, AMB showed higher μTBS than did PBA and SBU for both the adhesive strategies (p < 0.00001; Table 3).

After one year of water storage, there was a significant drop in the μTBS for all experimental groups (p < 0.00001; Table 3). However, some differences were observed between the groups. Regarding the adhesives, no significant difference in μTBS was observed when the dentin was kept wet (p > 0.05; Table 3). However, a significant difference was observed between the adhesives evaluated when the dentin was kept dry or oversaturated (p = 0.0001; Table 3).

For dry dentin, no significant difference was observed among the adhesives when the SE strategy was applied. However, AMB and SBU showed higher μTBSs than did PBA when dentin was kept dry and ER mode was employed (p < 0.00001; Table 3). On oversaturated dentin, AMB showed higher μTBS than did PBA and SBU used in both strategies. PBA also showed better μTBS than did SBU for oversaturated dentin (p < 0.00001; Table 3).

### Silver Nitrate Uptake (SNU)

Only the triple cross-product interaction was significant (adhesive vs moisture level vs storage time, p = 0.0001; Table 4). In addition, the main factors of adhesive (p = 0.0001), moisture level (p = 0.01), and storage time (p = 0.0001) were significant. The factor adhesive strategy was not significant (p = 0.62).

**Table 4:** Means and standard deviations of silver nitrate uptake (%) for all experimental groups

Moisture level	Time	AMB		PBA		SBU	
		ER	SE	ER	SE	ER	SE
Dry	24 h	6.3 ± 1.8 a,b	6.9 ± 2.1 a,b	14.1 ± 2.4 c,d	7.3 ± 2.7 b	15.7 ± 2.7 d	8.6 ± 2.7 b
	1 year	11.3 ± 2.8 c	12.6 ± 3.1 c	20.4 ± 4.2 e	13.2 ± 2.5 c	19.3 ± 3.7 e	15.6 ± 2.9 d
Wet	24 h	5.5 ± 1.7 a	5.2 ± 1.6 a	7.8 ± 1.8 b	8.6 ± 1.6 b	8.6 ± 2.5 b	7.4 ± 1.6 b
	1 year	11.4 ± 2.1 c	12.9 ± 2.6 c	14.0 ± 2.5 c,d	14.9 ± 3.1 c,d	15.8 ± 2.3 d	15.2 ± 2.2 d
Oversaturated	24 h	6.4 ± 1.9 a,b	5.3 ± 1.9 a	8.6 ± 1.7 b	8.6 ± 1.6 b	14.7 ± 1.8 c	14.2 ± 1.6 c
	1 year	12.4 ± 2.6 c	11.6 ± 2.3 c	15.6 ± 2.8 d	15.2 ± 2.7 d	30.7 ± 2.4 f	30.4 ± 1.9 f

Different letters indicate statistically different means (four-way ANOVA and Tukey’s test; p < 0.05).

After 24 h, a significant difference in the SNU was observed among all the adhesives and moisture levels evaluated. AMB had lower SNU values than did PBA and SBU at all moisture levels (p = 0.0001; Table 4). PBA also showed lower SNU values than did SBU on oversaturated dentin (p = 0.0001; Table 4).

After one year of water storage, there was a significant increase in the SNU values for all experimental groups (p = 0.0001; Table 4). However, it was not the same for all adhesives evaluated at all the moisture levels. When the dentin was kept dry or wet, AMB showed lower SNU values than did SBU (p = 0.001; Table 4). PBA showed intermediate values. When the dentin was overwet, AMB showed lower SNU values than did PBA and SBU (p = 0.0001; Table 4).

### SEM Evaluation of Hybrid Layer Morphology

**Fig 3:**
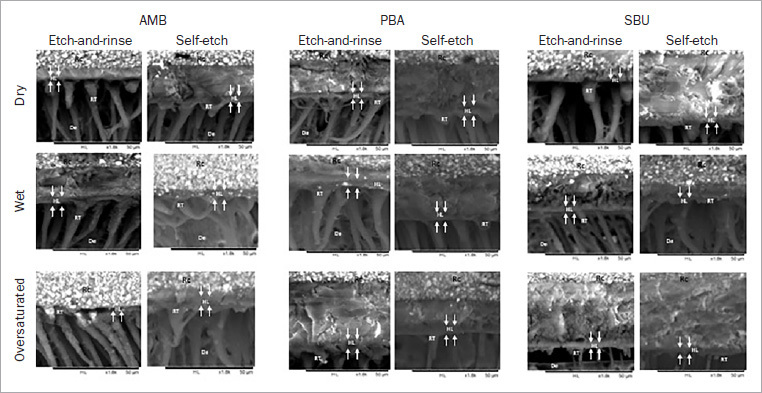
Representative SEM images (1800X) of hybrid-layer morphology for all groups. In general, independent of the universal adhesive and adhesive strategy, wet and dry dentin surfaces showed a more regular, uniform hybrid layer with formation of longer resin tags (RT). In contrast, when SBU was applied to oversaturated dentin, incomplete or short resin tags formed, especially when applied in SE mode (white arrows). Co: resin composite; AL: adhesive layer; HL: hybrid layer; RT: tesin tag; De: dentin.

Representative SEM images of the hybrid layer morphology produced by all the different groups at the immediate time point are shown in Fig 3. On the dry and wet dentin surfaces, all adhesives showed a hybrid layer with greater integrity and the presence of longer and more numerous resin tags. For oversaturated dentin and both strategies, AMB and PBA demonstrated a greater number of tags compared with SBU, which showed only a few needle-like resin tags, mainly when applied in SE mode.

### Degree of Conversion (DC)

The double cross-product interaction was significant (adhesive vs moisture level; Table 5; p < 0.0001). No significant difference in DC was observed between the adhesives when the dentin was kept wet (p > 0.05; Table 5). However, on dry dentin, AMB and SBU showed higher DC than did PBA (p < 0.0001; Table 5). On oversaturated dentin, AMB showed the highest DC compared with the other universal adhesives (p < 0.0001; Table 5). PBA also showed higher DC than did SBU on oversaturated dentin (p < 0.0001; Table 5).

**Table 5:** Degree of conversion (%) for all experimental group (*)

Moisture level	AMB	PBA	SBU
Dry	72.1 ± 2.3 a	66.5 ± 3.3 b	70.1 ± 2.9 a
Wet	72.5 ± 2.5 a	70.7 ± 2.7 a	69.3 ± 2.1 a
Oversaturated	69.4 ± 3.1 a	66.8 ± 2.9 b	58.6 ± 1.4 d

Different letters indicate statistically different means (two-way ANOVA and Tukey’s test; p < 0.05).

## DISCUSSION

During the last decade, universal adhesives have been introduced on the market to minimize the number of steps and reduce technique sensitivity given different degrees of dentinal surface wetness.^3,13,14^ However, only the immediate results have been evaluated.^3,13,14^ To the best of our knowledge, this is the first study to evaluate the bonding performance of several universal adhesives when applied to wet, dry, or oversaturated dentin at 24 h and 1 year, following storage of dentin in water.

It is known that demineralized dentin needs to be kept moist^49^ to maintain interfibrillar porosity for resin monomer infiltration and to allow high immediate bond strengths when ER mode is applied.^22,27,32,35,41^ When phosphoric acid-etched dentin was kept wet, this preserved the nanospaces between collagen fibrils^23^ into which the adhesive monomers diffused to envelop the collagen fibrils before polymerization. The higher infiltration rate of resin monomers into wet phosphoric acid-etched dentin might explain the adequate bonding performance of the universal adhesives in this study when the dentin remained wet in ER mode. On the other hand, several manufacturers have indicated that when applied in the ER strategy, universal adhesives can be used in wet or dry dentin because universal adhesives are less sensitive to moisture in the wet or dry range. These results are not new, since several in-vitro^6,32,35,41^ and in-vivo studies^2,7,16^ have shown no significant difference when universal adhesives were applied on dry or wet dentin.

However, interesting results were observed when dentin was kept wet or dry and universal adhesives were applied using the SE strategy. Studies on the previous generation of SE adhesives showed that it was not necessary to maintain dentin surface moisture prior to adhesive application.^1,5,40,42^ In this study, keeping the dentin surface wet or dry did not jeopardize the immediate bonding performance of all the adhesives evaluated.

This can be explained as follows: owing to the presence of 10-MDP universal adhesives, it is necessary for the adhesive to contain some amount of water.^43^ Although the exact composition of each universal adhesive evaluated is proprietary information, the water content of PBA is approximately 20%, whereas in SBU and AMB, it is between 10% and 15%. It seems that these amounts of water in the universal adhesives evaluated were not sufficient to jeopardize the immediate bond strengths when different universal adhesives were applied in SE mode under wet or dry conditions.

However, controversial results were found after one year of water storage when different universal adhesives were applied to oversaturated dentin, thus leading to rejection of the first and second null hypotheses. In fact, AMB showed higher μTBS and lower SNU values than did SBU and PBA, mainly after one year of water storage. Siqueira et al^32^ and Saeed et al^28^ reported that the composition of universal adhesives can compromise bonding performance on oversaturated dentin. This is mainly related to the photoinitiator system and resinous monomers in the adhesive formulation.

Usually, adhesives contain camphoroquinone (CQ) and an amine initiator to induce adequate polymerization.^43^ Although CQ is the most frequently employed photoinitiator in dentistry, it is hydrophobic and thus antagonistic to adhesive solutions containing hydrophilic components, which are required for interaction with tooth substrates. This hinders adequate adhesive infiltration into the oversaturated dentin surface.^50^ In reality, several studies have shown that adhesives containing a hydrophilic photoinitiator produced better results than CQ-containing adhesives in terms of polymerization efficiency and bond strength to dentin.^15,17^

Furthermore, SBU used only the CQ/amine system, whereas AMB contained a reduced amount of CQ. This is due to the presence of a more hydrophilic photoinitiator in AMB. As shown by Siqueira et al^32^ and in agreement with the results of this study, the in-situ DC of AMB was not compromised when this adhesive was used on a water-saturated dentin substrate. Those authors used a more hydrophilic photoinitiator to reduce the incompatibility of the hydrophilic-rich phase promoted by the hydrophobic form of CQ. Consequently, promoting better interaction of AMB on an oversaturated dentin substrate,^9,32^ as observed in the results of this study, led the authors to reject the third hypothesis. Unfortunately, manufacturers do not commonly list all the photoinitiators used in their products, which is the case for PBA. However, the DC was impaired for PBA when the dentin was oversaturated, indicating that a more hydrophobic photoinitiator should be used in this adhesive. Further studies are needed to confirm this hypothesis.

Regarding the monomer composition, SBU and PBA contain high-molecular-weight monomers, such as bisphenol A-glycidyl methacrylate (bis-GMA) and SBU contains polyalkenoic acid copolymer. The presence of a large amount of water, as in the case of oversaturated dentin, can induce phase separation of methacrylate adhesives, thus limiting the infiltration and consequently inhibiting the formation of a mechanically and structurally adequate resin-dentin interface.^33^ On the other hand, AMB contains UDMA instead of bis-GMA as the main monomer. Despite its comparable molecular weight, UDMA is less viscous and more flexible than bis-GMA.^43^

In the case of the polyalkenoic acid copolymer present in SBU, it is well known that this monomer does not dissolve well in the adhesive solution. Hence, a separate phase produced many globules within the polymer of the adhesive layer.^44^ Therefore, this may jeopardize the DC and consequently the bonding properties to oversaturated dentin. Indeed, the results of this study showed the presence of water bubbles and a significant increase in SNU when SBU was used on oversaturated dentin in comparison with SBU on wet dentin, or even when compared to AMB and PBA adhesives.

In contrast, no phase separation signs or bubbles were observed in the hybrid layer of PBA (Fig 2), even on oversaturated dentin. PBA does not contain polyalkenoic acid copolymers. In addition, according to the manufacturer, PBA has balanced hydrophobic and hydrophilic properties to ensure bonding at various moisture levels. This is mainly related to the use of the new liquid bifunctional acryl crosslinker (active-guard technology), which prevents undesired phase separation due to differences in surface wetness, as previously observed by Kumagai et al^13^ and Latta and Radniecki.^14^ This explains the better results shown by PBA compared to SBU.

Unfortunately, independent of moisture level, a significant decrease in μTBS and an increase in SNU for many experimental groups were observed after 1 year of water storage vs the immediate results. Although the exact mechanism that causes degradation of the hybrid layer is not yet completely understood, the first stage of biodegradation involves the extraction of poorly polymerized resins. This allows water to infiltrate into the dentin matrix within the hybrid layer.^29,34^ If the solvent and water are retained within the adhesive resin, they can severely compromise the structural integrity of the hybrid layer, thereby reducing its mechanical properties.^11,26^

Additionally, water softens the polymer network and reduces the frictional forces between polymeric chains.^8,26^ Unreacted monomers trapped in the polymer network are released into the surrounding area. This creates new channels through which even more water diffuses, resulting in a self-perpetuating process. As a consequence, the previously resin-infiltrated collagen matrix becomes exposed and vulnerable to attack by host-derived proteolytic enzymes,^38^ thus leading to a significant reduction in μTBS and an increase in the SNU for the majority of experimental groups, as observed in this study.

An exception was observed in the μTBS for all universal adhesives when applied to dry dentin using the SE strategy. This indicates that the amount of water in the adhesive bottle is sufficient to perform a strong chemical interaction with the dentin surface, and consequently maintain the longevity of bonding to dentin, in agreement with the results observed by Saeed et al.^28^

Universal adhesives have been advocated as a less sensitive bonding technique owing to the presence of 10-MDP and optimization of its water content. However, it appears that these products do not cover the entire moisture spectrum. In this study, not all the universal adhesives evaluated showed good results in terms of bond strength and silver nitrate uptake, particularly when oversaturated dentin was evaluated. Independent of dentin wetness, these new materials undergo degradation processes that occur when hydrophilic monomers are included in the composition of universal adhesives. Thus, the preferable condition for applying universal adhesives in terms of moisture dentin and adhesive strategy seems to be dry dentin associated with the SE strategy. Future studies should focus on the use of adhesives with more hydrophobic properties.

## CONCLUSION

Different universal adhesives applied in the etch-and-rinse and self-etch modes showed similar bonding results when applied to wet and dry dentin. However, the behavior of oversaturated dentin was dependent on the universal adhesive. Independent of the moisture level and the universal adhesive evaluated, significant degradation of the bonding properties occurred after 1 year of water storage, with the exception of universal adhesives applied in SE mode on dry dentin.

## Clinical relevance:

Universal adhesives are less sensitive to dentin wetness (dry or wet). However, the results for oversaturated dentin were material dependent. After 1 year of water storage, less pronounced degradation of the bonding properties occurred only when universal adhesives were applied to dry dentin.
